# Impact of COVID-19 pandemic on asthma symptoms and management: A prospective analysis of asthmatic children in Ecuador

**DOI:** 10.1016/j.waojou.2021.100551

**Published:** 2021-06-05

**Authors:** Angélica M. Ochoa-Avilés, Cristina Ochoa-Avilés, Diana A. Morillo-Argudo, María José Molina-Cando, Claudia R. Rodas-Espinoza, Irina Chis Ster, Manolo P. Maestre Calderón, Augusto Maldonado G, Karen Arteaga Vaca, Alejandro Rodriguez, Alvaro A. Cruz, Natalia Romero-Sandoval, Philip J. Cooper

**Affiliations:** a Department of Biosciences, Faculty of Chemistry, University of Cuenca, Cuenca, Azuay, Ecuador; b Instituto de Ciencias da Saude, Universidade Federal da Bahia, Salvador, Bahía, Brazil; c School of Medicine, International University of Ecuador, Quito, Pichincha, Ecuador; d Faculty of Medicine, University of Azuay, Cuenca, Azuay, Ecuador; e Institute of Infection and Immunity, St George's University of London, London, UK; f Department of Pediatrics Hospital Vicente Corral Moscoso, Cuenca, Azuay, Ecuador; g Universidad San Francisco de Quito School of Medicine, Donald and Barbara Zucker School of Medicine at Hofstra/Northwell, Hospital General Docente de Calderón, Quito, Pichincha, Ecuador; h Hospital Regional Verdi Cevallos, Portoviejo, Manabí, Ecuador; i Fundação ProAR and Universidade Federal da Bahia, Salvador, Brazil

**Keywords:** Children, Asthma, COVID-19, Lockdown, Ecuador

## Abstract

**Background:**

Asthma affects up to 33% of children in Latin American settings. The ongoing COVID-19 pandemic has had a significant impact on access to and use of health services. We aimed to evaluate the impact of the COVID-19 lockdown on asthma exacerbations, medical facility visits, and use of asthma medications in children.

**Methods:**

We used data from a prospective cohort of 213 children aged 5–17 years in 3 Ecuadorian cities and analysed the impact of the COVID-19 lockdown on asthma. Outcomes (asthma exacerbations, emergency room [ER] visits, planned and unplanned outpatient visits, and use of inhaled corticosteroids and Beta-2 agonists) were analysed using repeated Poisson counts (ie, number of events per participant before and during the COVID-19 lockdown).

**Results:**

During compared to before lockdown: a) the number of asthma exacerbations remained constant (IRR, 0.87; 95% CI: 0.72–1.05; p = 0.152); b) outpatient visits (IRR 0.26, 95% CI 0.14–0.47, p < 0.001) declined 74% while ER visits declined 89% (IRR 0.11, 95% CI 0.04–0.32, p < 0.001); and c) there was no change in inhaled corticosteroids use (IRR 1.03, 95% CI 0.90–1.16, P = 0.699) while Beta-2 agonist use increased (IRR 1.32, 95% CI 1.10–1.58, P = 0.003).

**Conclusions:**

In a cohort of Ecuadorian children with asthma, health services attendance decreased dramatically after COVID-19 lockdown, but asthma exacerbations and use of inhaled corticosteroids were unchanged. Future analyses will address the question of the effect of SARS-CoV-2 infection on asthma exacerbations and control in this paediatric population.

## Background

Asthma is the most common children's chronic disease and is estimated to affect between 9% and 33% of children in Latin American settings.^1^, ^2^, ^3^, ^4^ The ongoing COVID-19 pandemic has had a significant impact on access to and use of health services.^5^^,^^6^ Data from US hospital records and online surveys of health-care providers have documented major reductions in emergency room (ER) visits,^7^ uptakes of follow-up visits, prescriptions, treatment adherence,^6^^,^^8^ and asthma control.^5^^,^^9^ Similarly, hospital records analysis in Japan revealed a decrease in asthma hospitalisations during the pandemic.^10^ Such reductions likely reflect the combined effects of stay-at-home orders, reassignment of health services, fear of contagion or reduction of severe asthma attacks. There are limited data on the effects of COVID-19 on children with asthma. A recent multicentre case-control analysis of asthmatic children attending outpatient visits showed improved asthma outcomes and control during the COVID-19 pandemic.^11^ To our knowledge, there are no prospective published studies of the effect of COVID-19 control measures on asthma symptoms and management. In the present analysis, we used data from an ongoing prospective study of asthma in children and adolescents in 3 large Ecuadorian cities to study the impact of the implementation of lockdown measures to control the COVID-19 pandemic in Ecuador on the risk of asthma exacerbations, planned and unplanned health facility visits, and use of asthma medications.

## Methods

### Study design, and context

We used data from a prospective cohort study of children aged 5–17 years old being done in 3 cities in Ecuador (Quito, Cuenca, and Portoviejo) to identify risk factors for asthma exacerbations. Quito and Cuenca are located in the Andean highlands at 2500 m altitude, while Portoviejo is located in the coastal plains below 100 m. In Ecuador, mandatory COVID-19 lockdown provisions were implemented for 6 months between March 14 and September 13, 2020, and included stay-at-home orders, school and university closures, mandatory use of masks in public places, social distancing, prioritisation of teleworking, mobility restrictions, and curfews. Many public hospitals were assigned exclusively to COVID-19 emergency care, and planned outpatient appointments were cancelled during the lockdown.

### Recruitment

Children and adolescents attending emergency rooms (ERs) in public hospitals with an acute asthma attack between April 2019 and February 2020 were invited to participate***.*** Baseline information on sociodemographic data, asthma symptoms, and management of the previous 12 months was collected within 3 weeks of initial ER attendance. After the initial ER visit, outpatient follow-up appointments (either with a paediatric pneumologist or a paediatrician, depending on availability) were scheduled by hospital staff. Each specialist prescribed maintenance treatment without any intervention from the research team.

The research staff performed monthly follow-ups for the next 12 months through face-to-face meetings or by telephone interviews with parents or guardians to register occurrence of asthma exacerbations and symptoms, attendances at healthcare facilities, and ongoing treatments. The research staff did not prescribe medications but did provide free medications when not available within the public hospital pharmacies but only in accordance with the hospital specialists' prescriptions. At the start of COVID-19 lockdown, all scheduled face-to-face meetings were changed to monthly telephone interviews.

### Clinical outcomes

**Fig. 1:**
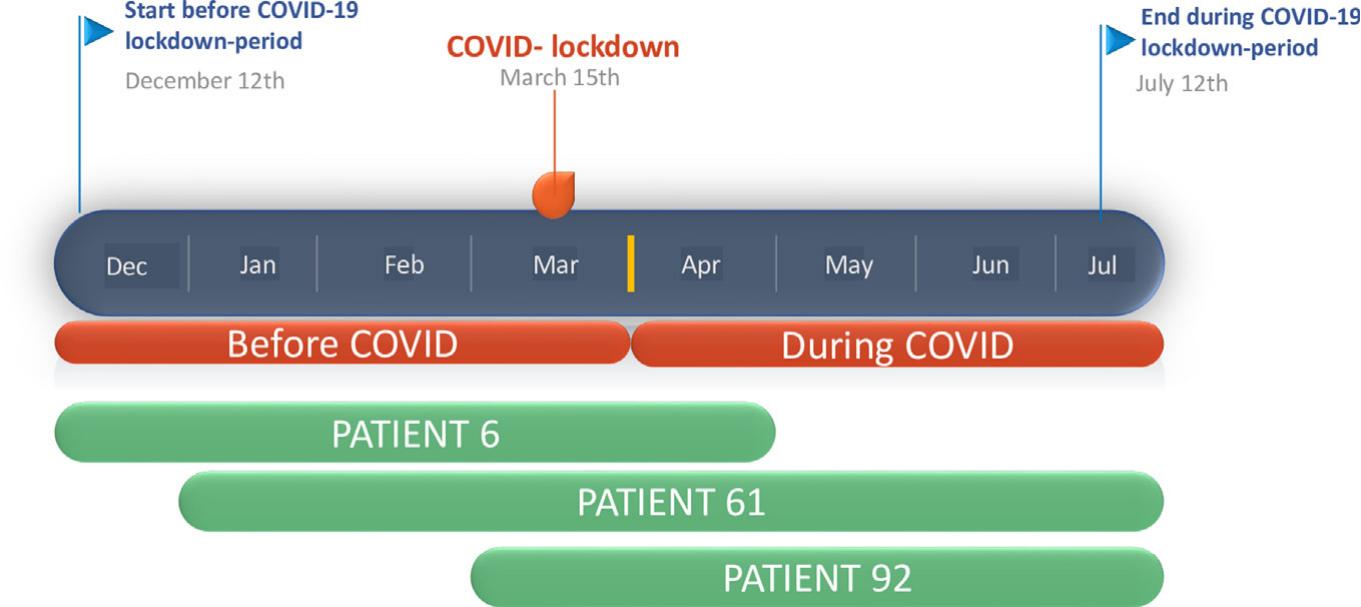
Example of follow-up time for three subjects used for estimation of incidence rate ratios of outcomes during versus after the COVID-19 lockdown

The clinical outcomes of primary interest were collected during the monthly follow-ups. The parents/guardians were interviewed to collect data on asthma exacerbations (including exact dates of occurrence), health facilities visits, and use on asthma medications in the previous month. Outcomes included: 1) asthma exacerbations defined as the number of parentally reported acute asthma attacks or wheezing episodes with respiratory distress; 2) number of ER visits; 3) number of outpatient visits including scheduled or unscheduled medical appointments for asthma (not including project-related activities for follow-up); 4) inhaled corticosteroid use defined by the number of follow-ups with inhaled corticosteroids usage in the previous month, and; 5) Beta-2 agonist usage defined by the number of follow-ups with Beta-2 agonist use in the previous month. All outcomes were evaluated before the COVID-19 lockdown (December 12, 2019 to March 31, 2020) and during the COVID-19 lockdown (April 1 to July 12, 2020). Fig. 1 shows examples of follow-up time included in the analysis for 3 study participants before and during COVID-19 lockdown. To assure the correct allocation of the exacerbations in the “before lockdown” or “during lockdown” periods, considering that monthly follow-up data was collected for the previous month, all data collected until March 31, 2020 were classified as “before lockdown period”. Next, all the dates of the reported exacerbations were checked, if an exacerbation was misclassified in the “before lockdown” or “during lockdown period” it was reallocated. Data were entered using ODK (Opendata kit v1.21.1 ODK Inc.) on mobile devices.

### Statistical analyses

Outcomes were analysed as repeated Poisson counts, assessed as the number of events per participant before and during COVID-19 restrictions, with associated offsets representing follow-up time potentially censored by the dates corresponding to 3 months before and during the lockdown. The associated offsets accommodate different periods of follow-up for each period for every child. Population averaged models were fitted for each outcome assuming exchangeable correlation structure and incidence rate ratios (IRRs) comparing monthly rates of events before and during lockdown (Table 1). Statistical significance was inferred by P < 0.05. Three-month average incidence rates of outcomes were also derived (Table 1). The analysis was performed with and without adjustment for age and sex, using Stata 12 (College Station, TX, USA).

**Table 1:** Incidence rates (IR) of outcomes during and after the COVID-19 lockdown and the respective incidence rate ratios (IRR)

Outcomes	During lockdown Total children × months follow up = 574			Before lockdown Total children × months follow-up = 712			During vs. Before lockdown	
	Total number of events	IR (95%CI)		Total number of events	IR (95% CI)		IRR (95% CI)	P value
		1-month	3-month		1-month	3-month		
Evaluations (follow-ups)	603	1.05 (0.97–1.14)	3.16 (2.92–3.43)	639	0.90 (0.83–0.97)	2.69 (2.50–2.90)	1.18 (1.05–1.32)	0.007
Asthma exacerbations	181	0.32 (0.27–0.36)	0.95 (0.82–1.09)	258	0.36 (0.32–0.41)	1.09 (0.96–1.23)	0.89 (0.66–1.16)	0.436
Emergency room visits	4	0.007 (0.003–0.018)	0.02 (0.01–0.06)	43	0.06 (0.04–0.08)	0.18 (0.13–0.24)	0.11 (0.04–0.32)	<0.001
Outpatient visits	13	0.023 (0.013–0.039)	0.07 (0.04–0.12)	61	0.085 (0.067–0.111)	0.26 (0.20–0.33)	0.26 (0.14–0.47)	<**0.001**
Inhaled corticosteroid use	221	0.39 (0.34–0.43)	1.14 (1.00–1.30)	260	0.37 (0.33–0.42)	1.12 (0.98–1.26)	1.02 (0.90–1.16)	0.705
Beta-2 agonist use	36	0.28 (0.24–0.32)	0.83 (0.71–0.96)	58	0.21 (0.18–0.25)	0.63 (0.53–0.74)	1.32 (1.10–1.59)	**0.002**

Incidence rates (IR) and incidence rate ratios (IRR) were estimated using Poisson regression models with offset indicating each child follow-up. P < 0.05 in bold. Evaluations - number of completed follow-ups; Asthma exacerbations – defined as acute attacks or asthma symptoms with respiratory distress; Emergency room visits - number of emergency room visits; Outpatient visits – number of scheduled or unscheduled visits; Inhaled corticosteroid (ICS) use - number of follow-ups with ICS usage in previous month; Beta_2_ agonist use - number of follow-ups with Beta_2_ agonist use in previous month.

### Ethical considerations

Study protocol and procedures were approved by the Ethics Committee of the Hospital Docente de Calderón (approval, CEISH-HGDC 2019- 001). Parents provided informed written consent, and children over 12 years provided informed minor consent. We provided inhaled corticosteroids to patients when prescribed by specialists and unavailable in hospital pharmacies including during the COVID-19 lockdown. During the monthly follow-ups, the research staff delivered basic non-pharmacological recommendations and contact information for hospital specialists where appropriate. In addition, a short educational video relating to COVID-19 and use of asthma medications was sent to all parents via social media, and parents were invited to a webinar on asthma care during the pandemic delivered by a paediatric pneumologist (MM), an immunologist (CR) and an infectious diseases physician.

## Results

We recruited 213 children in 3 Ecuadorian cities (Quito [n = 52], Cuenca [n = 98], and Portoviejo [n = 63]) with a mean age of 9.1 years (SD 2.9) of whom 48.4% were female, 94.4% self-identified as mestizo ethnicity (European-American indigenous), and 29.7% belonged to households with a monthly income below the Ecuadorian minimum wage (US$400).

### Baseline data

All subjects were recruited after attending an ER with an acute asthma attack. In the previous year before the asthma attack, 83.2% reported wheezing episodes, 17.4% had 4 or more episodes, and 29% had attended an ER more than twice. Although most participants had a doctor's diagnosis of asthma (69.7%), only 25.3% and 10.1% had used a Beta-2 agonist or inhaled corticosteroids, respectively, during the previous year prior to the asthma attack. At recruitment, 35.2% of the participants were using Beta-2 agonists, and 19.7% inhaled corticosteroids.

### Impact of the implementation of lockdown measures on asthma outcomes

**Fig. 2:**
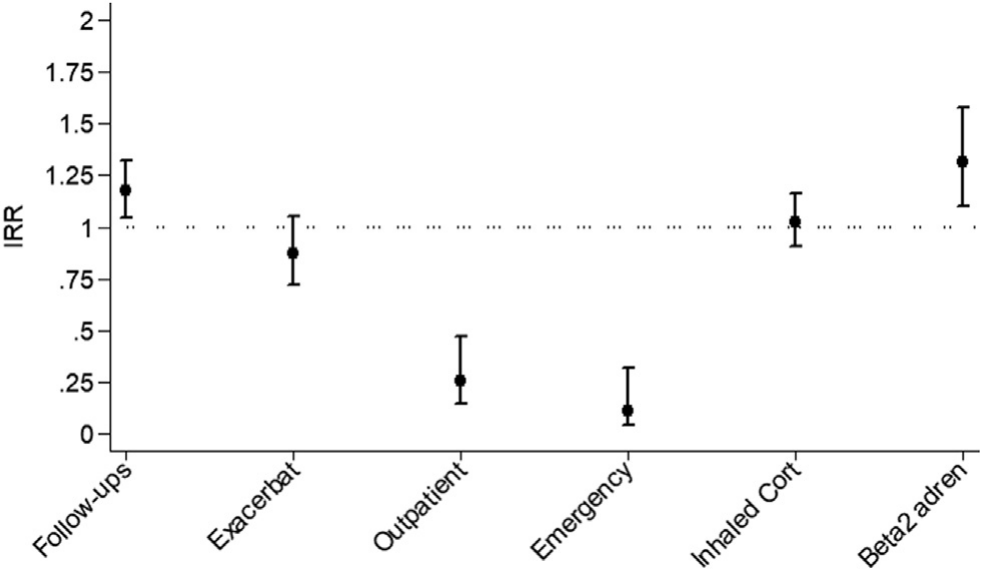
Incidence rate ratios (IRR) for outcomes comparing rates during versus before the COVID-19 lockdown. “Follow-ups”: the number of follow-ups completed; “Exacerbate”: acute asthma attacks or asthma symptoms with respiratory distress; “Outpatient” - all scheduled or unscheduled outpatient visits reported; “Emergency”: number of emergency room visits reported; “Inhaled Cort” - number of follow-ups with inhaled corticosteroids usage in the previous month; “Beta 2 adren” - number of follow-ups with Beta-2 agonist use in the previous month

IRRs for outcomes comparing rates during versus before COVID-19 lockdown are shown in Fig. 2 and Table 1. Adjusting for age and sex did not change the magnitude of the IRR estimates. There was no significant change in the number of reported asthma exacerbations during the lockdown measures compared to before (IRR, 0.87; 95% CI: 0.72–1.05; p = 0.152). However, outpatient visits (IRR 0.26, 95% CI 0.14–0.47, p < 0.001) declined 74%, while ER visits declined 89% (IRR 0.11, 95% CI 0.04–0.32, p < 0.001) during lockdown. Interestingly, there was no change in inhaled corticosteroids (IRR 1.03, 95% CI 0.90–1.16, P = 0.699), while Beta-2 agonist use increased by a third (IRR 1.32, 95% CI 1.10–1.58, P = 0.003). The overall number of monthly follow-ups increased during the lockdown (IRR 1.17, 95% CI 1.05–1.32, P = 0.007).

## Discussion

We have analysed a cohort of asthmatic children and adolescents in 3 Ecuadorian cities to determine the impact of the COVID-19 lockdown measures on the risk of asthma exacerbations, planned and unplanned health facility visits, and medication use. In Ecuador, strict measures were enforced for the duration of the lockdown follow-up period in all 3 cities. Our data showed marked declines in the use of health facilities but no impact on the number of asthma exacerbations during the implementation of lockdown measures to control the spread of COVID-19. Notably, the use of inhaled corticosteroids did not appear to be affected while that of Beta-2 agonists increased after the lockdown.

To our knowledge, there are no published data of the impact of COVID-19 lockdowns on asthma. Although we did not collect data on potential exposures to SARS-CoV-2 infections in the cohort, the lack of severe exacerbations requiring ER attendance could be interpreted as suggesting no link with SARS-CoV-2 infection. Available data indicate that asthma is not associated with an increased risk of COVID-19 disease in adults,^12^ except perhaps those with severe disease,^13^ but there is even less information on the relationship between COVID-19 and paediatric asthma.^7^

Control measures during lockdown likely would have reduced exposures to respiratory viruses^11^ that have been associated with 80% of asthma exacerbations in children, with rhinoviruses being particularly important.^14^ Several studies have shown rhinovirus infections to be linked to asthma exacerbations in children and adolescents in tropical regions of Latin America,^15^^,^^16^ although other respiratory viruses such as influenza and respiratory syncytial virus (RSV) may also have a role.^17^ Hospital admissions for asthma in Ecuador tend to vary by region and month with peak admissions in July through September in the coastal region (Portoviejo) and March through June in the highlands (Quito and Cuenca),^18^ probably reflecting regional differences in climate and circulation of respiratory viruses. To our knowledge, there are no published data on seasonality of rhinoviruses or regional differences in the seasonal circulation of other relevant respiratory viruses within Ecuador; at a country level, RSV appears to peak in March and influenza in December–January but with marked inter-year variation.^19^ Given the seasonal variations in asthma hospitalizations, an increase in asthma attacks during the lockdown period (from March 15) might have been expected in the two highland study centers but could have been masked by a probable decline in the circulation of respiratory viral infections, the main cause of asthma exacerbations,^14^ as a consequence of the greater social isolation during the COVID-19 lockdown.

Our findings are consistent with a recent international case-control study showing declines in ER visits among children with asthma during the COVID-19 pandemic.^11^ A generalised fear of COVID-19 might have led children with acute exacerbations to be managed at home rather than in ERs, explaining the dramatic fall (89%) in ER attendances and outpatient visits during lockdown. We did not observe changes in risk of asthma exacerbations during lockdown. In contrast, a recent case-control study examining the effects of the pandemic on several indicators of disease control in children with asthma attending outpatient facilities in 15 countries, showed a decline in asthma attacks and hospitalisations during the pandemic, although no account was taken of potential effects of timing and strictness of lockdown measures on these indicators.^11^ We did not assess the severity of symptoms in our study, and it is quite possible that there was a decline in severity rather than the frequency of symptoms. Inhaled corticosteroid use in our study population was maintained unchanged during the lockdown, which would have maximised asthma control. It has been suggested that inhaled corticosteroids may enhance innate immunity to viral infections,^14^^,^^15^ reduce susceptibility to severe respiratory viral infections,^15^ and in the case of SARS-CoV-2, downregulate virus angiotensin-converting enzyme-2 receptor (ACE2) expression in the airways.^16^ The increase in Beta-2 agonist use would indicate that patients did experience symptoms during lockdown – a previous study reported an increase in the use of Beta-2 agonists with worsening symptoms and delays in doctor visits.^20^

In conclusion, our data from a prospective study of paediatric asthma in Ecuador that compared asthma exacerbations, use of health services, and medication use before and during COVID-19 lockdown, showed dramatic reductions in the use of health services but no impact on the number of asthma exacerbations and use of inhaled corticosteroids. Future analyses of the cohort will address the question of the effect of SARS-CoV-2 infection on asthma exacerbations and control in this paediatric population.

## Abbreviations

ACE2, angiotensin-converting enzyme-2; ER, emergency room; IRR, incidence rate ratios; RSV, respiratory syncytial virus.

## Funding

This study forms part of the Asthma ATTACK study, a collaboration between the Universidad Internacional del Ecuador, Universidad de Cuenca, Universidad del Azuay, Universidade Federal da Bahia, Association ProAR, and St George's University of London, and was funded through the 10.13039/501100000272NIHR Global Health Research Group at 10.13039/501100012620St George's University of London by the 10.13039/501100000272National Institute of Health Research, UK (grant 17/63/62) using Official Development Assistance (ODA) funding.

The funding sources had no involvement in study design, data collection, analysis and interpretation of data, in the writing of the report, and in the decision to submit the article for publication.

## Submission declaration

All the authors have approved the final version, declare that it has not been published previously (except in the form of an abstract, a published lecture, or academic thesis) and it is not under consideration for publication elsewhere. Further, the authors confirm that, if accepted, it will not be published elsewhere in the same form, in English or in any other language, including electronically without the written consent of the copyright holder.

## Ethics approval

The study was approved by the Ethics Committee of the Hospital General Docente de Calderón, Quito, Ecuador (approval CEISH-HGDC 2019–001) and was conducted according to the ethical principles of the Declaration of Helsinki. All adolescents and their parents/guardians provided minor consent before participating.

## Authors contribution

PJC, NR, and AAC designed the study; ICS and AOA performed the statistical analysis; AMO, CO, ICS, and PJC drafted the manuscript; CO, DM, MJM, CR, MM, AM, KA, and AR contributed to data collection: All authors reviewed and approved the final manuscript.

## Declaration of competing interest

The authors report no competing interests.

## References

[1] Forno E., Gogna M., Cepeda A. (2015). Asthma in Latin America. Thorax.

[2] Weinmayr G., Forastiere F., Weiland S.K. (2008). International variation in prevalence of rhinitis and its relationship with sensitisation to perennial and seasonal allergens. Eur Respir J. Eur Respiratory Soc.

[3] Beasley R. (1998). Worldwide variation in prevalence of symptoms of asthma, allergic rhinoconjunctivitis, and atopic eczema: ISAAC. Lancet.

[4] Rosser F.J., Forno E., Cooper P.J., Celedón J.C. (2014). Asthma in hispanics. An 8-year update. Am J Respir Crit Care Med. American Thoracic Society.

[5] Oreskovic N.M., Kinane T.B., Aryee E., Kuhlthau K.A., Perrin J.M. (2020). The unexpected risks of COVID-19 on asthma control in children. J Allergy Clin Immunol Pract.

[6] Papadopoulos N.G., Custovic A., Deschildre A. (2020). Impact of COVID-19 on pediatric asthma: practice adjustments and disease burden. J Allergy Clin Immunol Pract.

[7] CDC COVID-19 Response Team (2020). Coronavirus disease 2019 in children - United States, february 12-april 2, 2020. MMWR Morb Mortal Wkly Rep.

[8] Taquechel K., Diwadkar A.R., Sayed S. (2020). Pediatric asthma healthcare utilization, viral testing, and air pollution changes during the COVID-19 pandemic. J Allergy Clin Immunol Pract.

[9] Freedman A., Tierney L. (2020). The Silver Lining to Coronavirus Lockdowns: Air Quality Is Improving.

[10] Abe K., Miyawaki A., Nakamura M., Ninomiya H., Kobayashi Y. (2021). Trends in hospitalizations for asthma during the COVID-19 outbreak in Japan. J Allergy Clin Immunol Pract.

[11] Papadopoulos N.G., Mathioudakis A.G., Custovic A. (2020). Childhood Asthma Outcomes during the COVID-19 Pandemic: Findings from the PeARL Multi-National Cohort.

[12] Halpin D.M.G., Faner R., Sibila O., Badia J.R., Agusti A. (2020). Do chronic respiratory diseases or their treatment affect the risk of SARS-CoV-2 infection?. Lancet Respir Med.

[13] Williamson E.J., Walker A.J., Bhaskaran K. (2020). Factors associated with COVID-19-related death using OpenSAFELY. Nature.

[14] Jackson D.J., Johnston S.L. (2010). The role of viruses in acute exacerbations of asthma. J Allergy Clin Immunol.

[15] Soto-Quiros M., Avila L., Platts-Mills T.A.E. (2012). High titers of IgE antibody to dust mite allergen and risk for wheezing among asthmatic children infected with rhinovirus. J Allergy Clin Immunol.

[16] Ardura-Garcia C., Vaca M., Oviedo G. (2015). Risk factors for acute asthma in tropical America: a case-control study in the City of Esmeraldas, Ecuador. Pediatr Allergy Immunol.

[17] Jartti T., Bønnelykke K., Elenius V., Feleszko W. (2020). Role of viruses in asthma.

[18] Instituto Nacional de Estadística y Censos. Camas Y Egresos Hospitalarios [Internet]. [cited 2021 Mar 9]. Available from: https://www.ecuadorencifras.gob.ec/camas-y-egresos-hospitalarios/.

[19] Caini S., de Mora D., Olmedo M. (2019). The epidemiology and severity of respiratory viral infections in a tropical country: Ecuador, 2009–2016. J Infect Public Health.

[20] Patel M., Pilcher J., Hancox R.J. (2015). The use of β2-agonist therapy before hospital attendance for severe asthma exacerbations: a post-hoc analysis. NPJ Prim Care Respir Med.

